# Prevalence, awareness, and treatment of chronic kidney disease among adults in Yunnan Province, China: findings from the 2023 chronic disease and risk factors surveillance

**DOI:** 10.3389/fpubh.2025.1685691

**Published:** 2025-10-15

**Authors:** Ying Shao, Yunyuan Fan, Jiao Gao, Haorong Meng, Meixian Wang, Qingping Shi, Yang Chen

**Affiliations:** ^1^Department for Chronic and Non-communicable Disease Control and Prevention, Yunnan Center for Disease Control and Prevention, Kunming, China; ^2^Department for Chronic and Non-communicable Disease Control and Prevention, Xishuangbanna Dai Autonomous Prefecture Center for Disease Control and Prevention, Xishuangbanna, China; ^3^School of Public Health, Kunming Medical University, Kunming, China

**Keywords:** chronic kidney disease, prevalence, awareness, treatment, distribution

## Abstract

**Background:**

Based on data from the seventh China Chronic Diseases and Risk Factors Surveillance (CCDRFS) conducted in Yunnan Province in 2023, this study aimed to provide province-wide estimates of the prevalence, awareness, and treatment of chronic kidney disease (CKD) and to describe their distribution.

**Methods:**

This provincial-level representative cross-sectional study included 6,231 adults sampled from 10 county- or district-level surveillance sites in Yunnan Province, Southwest China. All participants were subject to questionnaires, physical examinations, and laboratory tests. CKD-EPI equation was used to estimate glomerular filtration rate (eGFR) based on serum creatinine.

**Results:**

A total of 6,231 adults with measurements of eGFR and urine albumin were included in this study. The weighted prevalence of CKD was 13.1% (95% CI: 10.9–15.6%) overall, 12.7% (95% CI: 10.1–15.8%) among males, and 13.5% (95% CI: 10.2–17.6%) among females. Higher prevalence of CKD was observed in subgroups characterized by older age, lower education levels, being divorced/widowed/separated, and the presence of comorbidities (hypertension, diabetes, and hyperuricemia). Among participants with CKD, 61.5, 34.2, 3.2, 0.7, 0.2, and 0.1% were classified into stage G1, G2, G3a, G3b, G4, and G5, respectively. Overall CKD awareness and treatment rates were 3.0% (95% CI: 1.5–5.8%) and 1.0% (95% CI: 0.4–2.2%). Among individuals aware of their CKD status, 11.5% (95%CI: 6.5–19.9%) were receiving treatment. Participants with stage G4 or G5 demonstrated higher awareness and treatment. CKD patients with comorbid hypertension had higher awareness, while underweight CKD patients had higher treatment rates.

**Conclusion:**

The estimated CKD prevalence among adults in Yunnan Province in 2023 exceeded the national average (8.2%). CKD awareness and treatment rates were critically low. Targeted early screening programs or community-based interventions should be prioritized as urgent public health initiatives to improve disease recognition and treatment adherence for CKD prevention and management in China’s less developed regions.

## 1 Introduction

Chronic kidney disease (CKD) is defined as abnormalities in kidney structure or function persisting for over 3 months (1). Its primary manifestations include kidney damage and reduced kidney function. Characterized by high prevalence, substantial healthcare costs, and significant comorbidity risks—including severe cardiovascular and cerebrovascular diseases—CKD can lead to serious outcomes such as death and disability (2).

According to research led by the Global Kidney Health Atlas of the International Society of Nephrology (ISN-GKHA), the median prevalence of CKD across 167 countries worldwide is 9.5% (3). CKD was associated with 41.5 million disability-adjusted life years (DALYs). It is estimated that there are approximately 697 million CKD patients globally, and the prevalence of CKD has increased by 40% over the past 30 years (4).

The disease burden of CKD in China is also substantial. In 2018, the prevalence among Chinese residents was 8.2% (5). The age-standardized incidence of CKD in China was 163.74 per 100,000 population, with an age-standardized mortality of 10.84 per 100,000. Notably, the standardized incidence exhibited an upward trend between 1990 and 2021 (6).

Patients with end-stage renal disease (ESRD) require renal replacement therapy for survival, imposing substantial economic costs on families and society. Consequently, the prevention and treatment of CKD crucially depend on early detection, diagnosis, and intervention to reduce progression to ESRD.

Despite its high prevalence, CKD awareness remains relatively low compared to common chronic non-communicable diseases such as hypertension and diabetes (7, 8). This low awareness contributes to suboptimal treatment rate, thereby elevating the risk of ESRD progression.

Currently, published data on the prevalence, awareness, and treatment of CKD in Yunnan Province and across China remain scarce. Utilizing data from the seventh round of the China Chronic Disease and Risk Factor Surveillance (CCDRFS) conducted in Yunnan Province in 2023, this study provides more recent estimates of the prevalence, awareness, and treatment of CKD within the region. Pioneering in its scope, this study is the first to disclose the prevalence and distribution patterns of chronic kidney disease across underdeveloped provinces in Western China, based on a robust, provincially representative sample. The findings aim to inform future strategies for the prevention and management of CKD in less-developed areas.

## 2 Methods

### 2.1 Survey design and participants

This study is based on primary research conducted as part of the seventh CCDRFS in Yunnan. The CCDRFS is a cross-sectional, provincial representative survey, in 2023. Ten counties/districts were selected as monitoring sites from the 129 counties and districts in Yunnan Province. Site selection considered socio-economic development, demographic structure, while also adhering to principles of cost-effectiveness and sampling feasibility. This approach ensured provincial-level representativeness in terms of population composition, urbanization, education level, and birth and mortality rates (9).

Study participants comprised residents from the CCDRFS surveillance sites in Yunnan. Inclusion criteria were: ① age ≥ 18 years and ② permanent residency (residing in the county/district for ≥6 months within the past year). Pregnant women and individuals with cognitive or mental disorders, severe illnesses, or disabilities potentially affecting survey participation were excluded.

### 2.2 Data collection

Sampling was conducted by a multi-stage cluster random design across all Surveillance site. In the first two stages, three townships or streets and six administrative villages or neighborhood committees were systematically selected based on population size ranking. n the subsequent third and fourth stages, a total of 270 households were randomly selected from six residential clusters for survey inclusion. All residents within the selected households who met the inclusion criteria were enrolled as survey respondents. All investigators received standardized training and collected data using an electronic questionnaire system.

The CCDRFS collected data through three primary methods: standardized questionnaires, physical measurements, and laboratory tests. The face-to-face questionnaire was used to collect demographic characteristics, individual and family disease histories, behavior and lifestyle habits, as well as mental health indicators, etc. The physical measurements encompassed height, weight, waist circumference, blood pressure, and grip strength. Anthropometric measurements were obtained following a standardized protocol (10). Laboratory testing procedures were as follows: a 10-mL sample of fasting venous blood was collected from all participants for the quantification of hemoglobin, fasting blood glucose, glycated hemoglobin, total cholesterol, triglycerides, high-density lipoprotein cholesterol, low-density lipoprotein cholesterol, uric acid, creatinine, potassium, albumin, and total protein. Participants without a history of diabetes underwent an oral glucose tolerance test using 75 g of anhydrous glucose; a 2-mL venous blood sample was obtained 2 h after glucose administration to measure postprandial blood glucose levels. Additionally, a 5-mL morning urine sample was collected from all participants for the analysis of urinary creatinine, microalbuminuria, sodium, and potassium. All examinations, except for the questionnaire, were performed in the morning after an overnight fast.

Blood glucose and hemoglobin were analyzed at certified monitoring laboratories that had passed standardized performance verification. All other biochemical indicators were measured at a central laboratory accredited by the Chinese Center for Disease Control and Prevention and holding all relevant certifications. The following analytical methods were applied: plasma glucose was measured on-site either by the hexokinase method or glucose oxidase method; hemoglobin was determined on-site using the HemoCue® system; glycated hemoglobin was quantified via high-performance liquid chromatography (HPLC); total cholesterol was assessed with the cholesterol oxidase-aminoantipyrine phenol (CHOD-PAP) method; triglycerides were analyzed using the phosphoglyceric oxidase method; high-density and low-density lipoprotein cholesterol were both measured via homogeneous enzyme colorimetric assays; uric acid was determined by the uricase-peroxidase method; both blood and urinary creatinine were analyzed using an enzyme-coupled sarcosine oxidase assay; total serum protein was measured with the biuret method; serum albumin was quantified via the bromocresol green method; urinary microalbumin was determined by immunoturbidimetry; and blood potassium, urinary potassium, and urinary sodium were analyzed using ion-selective electrode methods.

### 2.3 Assessment criteria

The diagnosis and staging of chronic kidney disease (CKD) adhered to the 2024 Kidney Disease Outcomes Quality Initiative (KDIGO) Clinical Practice Guideline for Evaluation and Management of Chronic Kidney Disease. CKD was defined as an estimated glomerular filtration rate (eGFR) < 60 mL/min/1.73 m^2^ or a urine albumin-to-creatinine ratio (UACR) ≥ 30 mg/g. For patients meeting these diagnostic criteria, stages Grade 1 to Grade 5 are defined based on eGFR [ml/min/1.73 m^2^] as follows: (1) G1: ≥90, G2: 60–89, G3a: 45–59, G3b: 30–44, G4: 15–29, G5: <15. The eGFR was calculated using the Chronic Kidney Disease Epidemiology Collaboration (CKD-EPI) 2021 creatinine equation based on serum creatinine (Scr) values for males and females, respectively (11): Due to the limitations inherent in the cross-sectional study design, serum creatinine and urinary albumin measurements were performed at a single time point without repeated assessment after 3 months. As a result, it was not possible to exclude some cases of reversible renal impairment or transient proteinuria, which may have led to an overestimation of the prevalence rates.

The prevalence of chronic kidney disease was defined as the proportion of individuals meeting diagnostic criteria within the surveyed population.

The CKD awareness was defined as the proportion of individuals meeting CKD diagnostic criteria and self-report a prior diagnosis of CKD by a medical institution.

The CKD treatment was defined as the proportion of individuals meeting CKD diagnostic criteria who self-report receiving any form of treatment for the disease, including angiotensin-converting enzyme inhibitors (ACEIs), angiotensin II receptor blockers (ARBs), glucocorticoid, immunosuppressant, or dialysis.

The treatment-awareness rate was defined as the proportion of individuals receiving kidney disease treatment among those aware of their CKD diagnosis.

Hypertension (12) was defined as systolic blood pressure (SBP) ≥ 140 mmHg and/or a diastolic blood pressure (DBP) ≥ 90 mmHg, and/or self-reported prior diagnosis of hypertension by a hospitals at township (community) level or higher.

Diabetes was defined as fasting plasma glucose (FPG) ≥ 7.0 mmol/L and/or a 2 h oral glucose tolerance test plasma glucose (OGTT-2 h) ≥ 11.1 mmol/L and/or self-reported prior diagnosis of diabetes by a hospitals at township (community) level or higher (13).

Weight status categories (underweight, normal weight, overweight, obesity) were determined based on body mass index (BMI) (14): BMI < 18.5 considered as underweight, BMI between 18.5 and 23.9 considered as normal weight, BMI between 24.0 and 27.9 considered as overweight, and BMI ≥ 28.0 considered as obesity.

Hyperuricemia was defined as a serum uric acid level >420 μmol/L measured under fasting conditions.

### 2.4 Statistical analysis

Statistical analyses were performed by SPSS 20.0’s complex samples module. Prevalence estimates were calculated using sample weights that incorporated the multistage sampling weight, the non-response weight, and the post-stratification weight is equivalent to direct standardization based on the 2010 China’s Sixth National Census population. The 95% CIs of the prevalence estimates were calculated using Taylor series linearization with finite population correction implemented. The *χ*^2^ test was used to compare rate distributions across populations.

### 2.5 Ethics statement

The study was approved by the Ethics Review Committee of the National Center for Chronic and Noncommunicable Diseases Control and Prevention (NCNCD), Chinese Center for Disease Control and Prevention (Approval No. 202305). The authors assert that all procedures contributing to this work comply with the Helsinki Declaration of 1975, as revised in 2008. Written informed consent was obtained from all participants.

## 3 Results

### 3.1 Study population

A total of 6,231 participants were included. The sample distribution was as follows: 44.6% male and 55.4% female; 28.3% urban residents and 71.7% rural residents; and 16.6% aged 18–39 years, 48.4% aged 40–59 years, and 35.0% aged ≥60 years, as shown in Table 1.

**Table 1:** General characteristics of the study population of Yunnan adult in 2023.

Characteristic	Urban		Rural		Total	
	No.	%	No.	%	No.	%
Total	1764	28.3	4,667	71.7	6,231	100.0
Gender						
Male	776	12.5	2004	32.2	2,780	44.6
Female	988	15.9	2,463	39.5	3,451	55.4
Age group, y						
18–39	288	4.6	747	12.0	1,035	16.6
40–59	784	12.6	2,234	35.9	3,018	48.4
≥60	692	11.1	1,486	23.8	2,178	35.0
Ethnicity						
Han	1,226	19.7	2,485	39.9	3,711	59.6
Yi	139	2.2	698	11.2	837	13.4
Other	399	6.4	1,284	20.6	1,683	27.0
Education						
Primary school or lower	827	14.0	3,015	48.4	3,887	62.4
Secondary and high school	781	12.5	1,295	20.8	2076	33.3
College or above	111	1.8	157	2.5	268	4.3
Marital status						
Never married	60	1.0	177	2.8	237	3.8
Married	1,560	25.0	3,940	63.2	5,500	88.3
Divorced or widowed	144	2.3	350	5.6	494	7.9
Hypertension						
Diagnosed	782	12.9	1783	29.3	2,565	42.2
No hypertension	957	15.7	2,562	42.1	3,519	57.8
Diabetes						
Diagnosed	288	4.8	455	7.7	743	12.5
No diabetes	1,424	24.0	3,772	63.5	5,196	87.5
BMI, kg/m^2^						
≥28.0	254	4.2	533	8.8	787	13.0
24.0–27.9	606	10.0	1,409	23.3	2015	33.3
18.5–23.9	801	13.2	2,170	35.9	2,971	49.1
<18.5	67	1.1	209	3.5	276	4.6
Hyperuricemia						
Diagnosed	359	6.0	777	12.9	1,136	18.8
No hyperuricemia	1,369	22.7	3,523	58.4	4,892	81.2

### 3.2 Prevalence of CKD

The overall prevalence of CKD among participants was 13.1% (95% CI: 10.9–15.6%). No statistically significant difference was observed between males (12.7%; 95% CI: 10.1–15.8%) and females (13.5%; 95% CI: 10.2–17.6%) (*χ*^2^ = 0.114, *p* > 0.05). Prevalence increased significantly with age (*χ*^2^ = 15.556, *p* < 0.001). Significant prevalence variations were also found across education levels (*χ*^2^ = 10.495, *p* < 0.001) and marital statuses (*χ*^2^ = 19.988, *p* < 0.001). Participants with chronic conditions (hypertension, diabetes, hyperuricemia) exhibited significantly higher CKD prevalence than those without (*p* < 0.05) (Table 2).

**Table 2:** Weighted prevalence of chronic kidney disease (CKD) by stage and ethnic group among adults in Yunnan Province, 2023.

Characteristic	Number of valid samples (N)	CKD prevalence (%, 95%CI)	CKD stage (%, 95%CI)					
			G1 or normal	G2	G3a	G3b	G4	G5
Total	6,231	13.1 (10.9–15.6)	61.5 (58.8–64.1)	34.2 (31.8–36.8)	3.2 (2.7–3.8)	0.7 (0.5–1.1)	0.2 (0.1–0.5)	0.1 (0.1–0.3)
Gender								
Male	2,780	12.7 (10.1–15.8)	56.7 (52.4–60.9)	38.7 (34.7–42.8)	3.2 (2.5–4.2)	1.0 (0.5–1.8)	0.2 (0.1–0.7)	0.2 (0.0–0.5)
Female	3,451	13.5 (10.2–17.6)	66.5 (63.4–69.4)	29.5 (26.8–32.4)	3.2 (2.5–4.1)	0.4 (0.2–0.7)	0.2 (0.1–0.5)	0.1 (0.0–0.3)
*χ* ^2^		0.114	6.314					
*p*-value		0.736	<0.001					
Age group, y								
18–39	1,035	7.3 (3.5–14.6)	87.5 (83.6–90.6)	12.1 (9.0–16.0)	0.2 (0.0–1.1)	0.0 (0.0–0.1)	0.2 (0.0–1.1)	0.0 (0.0–0.0)
40–59	3,018	11.1 (9.2–13.4)	57.5 (54.3–60.5)	40.7 (37.7–43.8)	1.5 (1.1–2.2)	0.2 (0.1–0.5)	0.0 (0.0–0.1)	0.1 (0.0–0.2)
≥60	2,178	28.8 (25.8–32.0)	15.9 (14.0–18.0)	66.5 (63.4–69.5)	12.9 (10.7–15.4)	3.2 (2.0–5.2)	0.9 (0.4–1.6)	0.6 (0.2–1.6)
*χ* ^2^		15.556	103.258					
*p*-value		<0.001	<0.001					
Township								
Urban	1764	14.4 (10.6–19.2)	66.2 (61.8–70.3)	30.0 (26.2–34.1)	2.9 (2.2–3.9)	0.7 (0.3–1.5)	0.2 (0.1–0.5)	0.0 (0.0–0.2)
Rural	4,667	11.7 (10.4–13.2)	56.6 (54.0–59.1)	38.7 (36.2–41.2)	3.5 (2.8–4.4)	0.8 (0.5–1.2)	0.2 (0.1–0.7)	0.2 (0.1–0.6)
*χ* ^2^		1.533	5.602					
*p*-value		0.216	<0.001					
Ethnicity								
Han	3,711	13.0 (10.7–15.6)	60.6 (57.2–64.0)	34.9 (31.8–38.1)	3.3 (2.7–4.2)	0.7 (0.4–1.3)	0.2 (0.1–0.5)	0.2 (0.1–0.5)
Yi	837	19.6 (6.4–46.4)	73.5 (64.6–80.8)	24.8 (17.9–33.3)	1.4 (0.8–2.4)	0.2 (0.1–0.5)	0.1 (0.0–0.3)	0.0 (0.0–0.0)
Other	1,683	11.7 (9.2–14.8)	60.5 (56.0–64.8)	35.0 (31.0–39.0)	3.3 (2.5–4.5)	0.8 (0.4–1.4)	0.3 (0.1–1.3)	0.1 (0.0–0.4)
*χ* ^2^		0.640	1.895					
*p*-value		0.462	0.077					
Education								
Primary school or lower	3,887	18.3 (15.4–21.6)	45.2 (42.1–48.4)	47.5 (44.4–50.6)	5.6 (4.6–6.9)	1.2 (0.7–1.9)	0.4 (0.2–0.7)	0.1 (0.0–0.4)
Secondary and high school	2076	11.2 (7.7–16.0)	69.8 (65.9–73.4)	27.6 (24.2–31.4)	1.9 (1.3–2.7)	0.4 (0.2–1.2)	0.1 (0.0–0.2)	0.2 (0.0–0.6)
College or above	268	3.9 (2.0–7.4)	81.5 (73.4–87.6)	17.4 (11.5–25.4)	0.5 (0.1–3.4)	0.2 (0.0–1.6)	0.4 (0.1–2.9)	0.0 (0.0–0.0)
*χ* ^2^		10.495	18.609					
*p*-value		*P*<0.001	<0.001					
Marital status								
Never married	237	4.7 (2.5–8.6)	87.4 (78.8–92.8)	11.1 (5.9–19.7)	0.7 (0.2–2.4)	0.4 (0.1–2.7)	0.5 (0.1–3.4)	0.0 (0.0–0.0)
Married	5,500	12.6 (10.4–15.2)	59.0 (56.2–61.7)	36.9 (34.3–39.6)	3.1 (2.5–3.8)	0.7 (0.4–1.2)	0.2 (0.1–0.3)	0.1 (0.0–0.4)
Divorced or widowed	494	37.1 (24.4–51.8)	33.2 (20.5–49.0)	52.9 (40.7–64.9)	11.1 (7.4–16.4)	1.5 (0.6–3.7)	0.7 (0.1–3.0)	0.6 (0.1–2.5)
*χ* ^2^		19.988	12.777					
*p*-value		<0.001	<0.01					
Hypertension								
Diagnosed	2,512	24.3 (21.3–27.6)	41.4 (37.7–45.1)	49.2 (45.6–52.8)	7.0 (5.6–8.6)	1.8 (1.0–3.0)	0.5 (0.2–0.9)	0.3 (0.1–0.9)
No hypertension	3,457	7.8 (5.2–11.5)	70.8 (67.7–73.7)	27.3 (24.5–30.3)	1.5 (1.1–2.0)	0.2 (0.1–0.5)	0.1 (0.0–0.6)	0.1 (0.0–0.2)
*χ* ^2^		35.729	52.499					
*p*-value		<0.001	<0.001					
Diabetes								
Diagnosed	731	29.1 (24.0–34.9)	51.1 (44.7–57.4)	40.5 (34.7–46.6)	5.5 (3.6–8.4)	1.8 (0.9–3.3)	0.4 (0.1–1.7)	0.7 (0.2–2.8)
No diabetes	5146	11.3 (9.0–14.1)	62.9 (60.0–65.7)	33.2 (30.6–35.9)	3.0 (2.5–3.6)	0.6 (0.3–1.1)	0.2 (0.1–0.5)	0.1 (0.0–0.2)
*χ* ^2^		42.942	6.637					
*p*-value		<0.001	<0.001					
BMI, kg/m2								
≥28.0	269	16.7 (12.1–22.5)	64.7 (57.5–71.3)	31.2 (25.2–38.0)	2.3 (1.5–3.7)	1.2 (0.5–2.6)	0.5 (0.1–2.4)	0.0 (0.0–0.0)
24.0–27.9	1993	14.7 (10.3–20.4)	54.9 (50.2–59.5)	41.2 (36.8–45.7)	3.4 (2.5–4.6)	0.4 (0.2–0.8)	0.1 (0.0–0.6)	0.0 (0.0–0.0)
18.5–23.9	2933	11.4 (8.7–14.7)	62.5 (58.8–66.0)	32.8 (29.6–36.2)	3.5 (2.7–4.5)	0.8 (0.4–1.7)	0.2 (0.1–0.4)	0.2 (0.1–0.6)
<18.5	269	8.6 (4.6–15.7)	79.6 (69.2–87.1)	16.7 (10.4–25.8)	2.5 (1.1–5.7)	0.0 (0.0–0.3)	0.6 (0.1–3.1)	0.5 (0.1–2.2)
*χ* ^2^		1.653	3.510					
*p*-value		0.181	<0.001					
Hyperuricemia								
Diagnosed	1133	18.4 (13.6–24.5)	51.5 (44.2–58.7)	40.7 (34.3–47.4)	5.3 (3.8–7.2)	1.7 (1.1–2.7)	0.5 (0.2–1.6)	0.4 (0.1–1.2)
No hyperuricemia	4839	11.4 (9.1–14.1)	64.6 (61.8–67.2)	32.3 (29.8–34.9)	2.6 (2.1–3.2)	0.4 (0.2–0.9)	0.2 (0.1–0.3)	0.0 (0.0–0.2)
*χ* ^2^		6.407	8.495					
*p*-value		0.011	<0.001					

Among CKD patients, 95.7% were classified as G1/G2 renal function, while G3a, G3b, G4, and G5 stages accounted for 3.2, 0.7, 0.2, and 0.1%, respectively. Stage distribution varied significantly by gender (*χ*^2^ = 6.314, *p* < 0.01) and exhibited strong age-dependency (*χ*^2^ = 103.258, *p* < 0.001): the proportion of G1/G2 stages decreased with advancing age, whereas G3a + stages increased progressively. Significant variations were also observed across township (*χ*^2^ = 5.602, *p* < 0.001), educational levels (*χ*^2^ = 18.609, *p* < 0.001), marital statuses (*χ*^2^ = 12.777, *p* < 0.001). Patients with hypertension, diabetes, overweight/obesity, or hyperuricemia showed significantly higher proportions of CKD G2 + stages compared to those without these comorbidities (*p* < 0.001; Table 2).

### 3.3 Awareness and treatment of CKD

Among CKD patients, the awareness was 3.0% (95% CI: 1.5–5.8%). Awareness varied significantly by renal function stage, with G4+ patients demonstrating higher awareness than G3- patients (*χ*^2^ = 6.977, *p* < 0.001). Hypertensive CKD patients also showed elevated awareness versus non-hypertensive patients (*χ*^2^ = 3.913, *p* = 0.048) (Table 3).

**Table 3:** Weighted prevalence, awareness, treatment, and treatment in awared patients among population with CKD, 2023 (%, 95%CI)

Characteristic	Number of valid samples (*N*)	Awareness	Treatment	Treatment in awared patients
Total	6,231	3.0 (1.5–5.8)	1.0 (0.4–2.2)	11.5 (6.5–19.9)
Gender				
Male	2,780	3.2 (1.1–9.1)	0.5 (0.2–1.4)	13.3 (6.2–26.4)
Female	3,451	2.7 (1.3–5.4)	1.4 (0.5–4.1)	8.2 (4.3–15.1)
*χ* ^2^		0.065	1.985	0.979
*p*-value		0.798	0.159	0.322
Age group, y				
18–39	1,035	0.0 (0.0–0.0)	0.0 (0.0–0.0)	0.0 (0.0–0.0)*
40–59	3,018	4.9 (1.7–13.3)	1.0 (0.2–4.1)	14.3 (6.4–28.9)*
≥60	2,178	3.0 (1.7–5.3)	1.5 (0.6–3.7)	12.7 (7.6–20.5)*
*χ* ^2^		1.343	0.803	3.865
*p*-value		0.258	0.444	0.044
Township				
Urban	1764	2.0 (0.4–9.1)*	0.4 (0.1–2.5)	13.3 (4.4–34.0)
Rural	4,667	4.2 (2.6–6.7)*	1.6 (0.7–3.9)	10.2 (6.1–16.4)
*χ* ^2^		0.830	1.987	0.205
*p*-value		0.362	0.159	0.651
Ethnicity				
Han	3,711	3.9 (1.8–8.2)	1.3 (0.6–3.2)	15.3 (7.8–27.6)
Yi	837	1.0 (0.2–5.0)	0.4 (0.0–3.3)	5.5 (1.7–16.0)
Other	1,683	1.3 (0.4–4.0)	0.2 (0.0–1.5)	5.0 (1.3–17.3)
*χ* ^2^		2.517	2.462	2.521
*p*-value		0.086	0.086	0.100
Education				
Primary school or lower	3,887	2.7 (1.5–4.9)	1.3 (0.5–3.4)	10.5 (6.1–17.6)
Secondary and high school	2076	3.7 (1.0–12.4)	0.6 (0.2–1.9)	14.5 (5.5–33.0)
College or above	268	0.0 (0.0–0.0)	0.0 (0.0–0.0)	0.0 (0.0–0.0)
*χ* ^2^		0.250	0.392	1.182
*p*-value		0.736	0.640	0.288
Marital status				
Never married	237	0.0 (0.0–0.0)	0.0 (0.0–0.0)	0.0 (0.0–0.0)
Married	5,500	3.1 (1.4–6.7)	0.9 (0.3–2.3)	12.5 (6.7–22.3)
Divorced or widowed	494	3.0 (1.0–5.8)	1.6 (0.4–7.0)	14.2 (5.1–33.5)
*χ* ^2^		0.239	0.311	0.622
*p*-value		0.757	0.728	0.480
CKD stage				
G1	166	2.9 (0.6–13.5)*	0.6 (0.1–4.5)*	4.9 (1.8–12.3)*
G2	266	2.6 (1.2–5.3)*	0.4 (0.1–1.1)*	12.8 (4.4–31.9)*
G3a	297	1.3 (0.6–3.0)*	0.2 (0.0–1.3)*	40.4 (22.2–61.7)*
G3b	54	1.6 (0.3–7.9)*	0.1 (0.0–0.4)*	56.5 (16.2–89.7)*
G4	20	15.0 (2.9–51.0)*	12.2 (1.7–52.1)*	62.4 (14.5–94.2)*
G5	8	42.6 (12.5–79.3)*	31.9 (8.1–71.3)*	75.0 (31.5–95.1)*
*χ* ^2^		6.977	24.464	6.639
*p*-value		*P*<0.001	*P*<0.001	*P*<0.001
Hypertension				
Diagnosed	566	4.1 (1.9–8.7)	1.1 (0.4–3.0)	17.3 (8.5–32.1)
No hypertension	245	1.3 (0.5–3.3)	0.8 (0.2–2.9)	5.7 (2.4–12.7)
*χ* ^2^		3.913	0.171	4.542
*p*-value		0.048	0.679	0.033
Diabetes				
Diagnosed	196	4.8 (1.0–19.6)	0.0 (0.0–0.0)	4.7 (1.6–13.4)
No diabetes	602	2.5 (1.4–4.2)	1.3 (0.6–2.9)	12.4 (6.7–21.8)
*χ* ^2^		0.678	1.466	2.617
*p*-value		0.410	0.226	0.106
BMI, kg/m^2^				
≥28.0	145	5.7 (1.2–22.2)	0.1 (0.0–0.7)	6.9 (2.7–16.2)
24.0–27.9	279	1.3 (0.5–3.6)	0.0 (0.0–0.1)	3.6 (1.6–8.0)
18.5–23.9	354	2.6 (1.3–5.2)	1.5 (0.6–4.1-)	16.8 (7.1–34.8)
<18.5	33	8.6 (1.9–31.0)	8.6 (1.9–31.0)	38.8 (15.2–69.1)
*χ* ^2^		1.627	13.634	5.301
*p*-value		0.193	*P*<0.001	0.003
Hyperuricemia				
Diagnosed	279	3.4 (1.7–6.7)	1.8 (0.6–4.9)	9.4 (4.5–18.6)
No hyperuricemia	532	2.8 (1.0–7.3)	0.6 (0.2–2.1)	12.6 (6.0–24.8)
*χ* ^2^		0.098	1.960	0.333
*p*-value		0.754	0.162	0.564

Among CKD patients, the self-reported treatment was 1.0% (95% CI: 0.4–2.2%). Treatment rates significantly differed by renal function stage, with G4 + patients demonstrating higher rates than G3- patients (*χ*^2^ = 24.464, *p* < 0.001). Underweight CKD patients showed higher treatment rates than normal/overweight-obese patients (*χ*^2^ = 13.634, *p* < 0.001) (Table 3).

Among CKD patients aware of their diagnosis, 11.5% (95% CI: 6.5–19.9%) reported current treatment. Treatment rates varied significantly by age (*χ*^2^ = 3.865, *p* = 0.044), renal function stage (*χ*^2^ = 6.639, *p* < 0.001). Hypertensive patients showed higher rates than non-hypertensive counterparts (*χ*^2^ = 4.542, *p* = 0.033), with underweight patients demonstrating higher rates than normal/overweight-obese patients (*χ*^2^ = 5.301, *p* = 0.003) (Table 3).

## 4 Discussion

The prevalence of CKD in Yunnan Province is higher than the national average and is on an upward trend. This study analyzed CKD prevalence, renal function staging, awareness, treatment, and epidemiological characteristics among Yunnan residents using surveillance data from over 6,000 adults in 10 surveillance sites in 2023. Results revealed a 13.1% CKD prevalence. Based on Yunnan’s Seventh National Census data (age ≥ 15 years), this corresponds to approximately 4.9 million affected individuals. According to the same diagnostic criteria for CKD, the prevalence in Yunnan Province is significantly exceeds China’s 2018 national prevalence (8.2%) (15). Yunnan’s CKD prevalence exceeds published rates for Henan Province (10.5%) (16), Guangzhou (12.1%) (17), and Zhejiang Province (9.9%) (18). However, it remains lower than the 25.8% prevalence reported for Pudong New Area in Shanghai (age 20–79 cohort) (19).

The global prevalence of chronic kidney disease (CKD) varies significantly across different regions. In high-income countries, including the United States (20), Australia (21), Netherlands, and Norway (22, 23), the estimated prevalence of CKD ranges between 10 and 13%. In contrast, low- and middle-income countries exhibit considerable disparities. For instance, Bolivia and Iran report relatively lower rates, between 5.5 and 6.3%, whereas China, Mongolia, India, Nepal, and Nigeria show markedly higher prevalence, ranging from 16 to 29.9% (24). Furthermore, the primary etiologies of CKD differ across regions with varying development levels and are not limited to hypertension and diabetes. In certain developing countries, up to 40% of CKD cases are attributable to non-traditional risk factors, such as infectious diseases (e.g., HIV and tuberculosis) (25), environmental toxin exposure (26), or unknown causes (27), rather than diabetes or cardiovascular conditions.

Compared to the other common chronic diseases in Yunnan Province, the prevalence of CKD is lower than that of hypertension (24.1% in 2018), but higher than that of diabetes (7.1% in 2018) ^28^. The CKD prevalence in 2023 has increased by 5.4% compared to in 2018 (7.7%) While the prevalence of hypertension and diabetes in Yunnan are both below the national average (5), the CKD prevalence exceeds it. This disparity may stem from two factors: First, hypertension and diabetes are key drivers in the development of chronic kidney disease (CKD) and have been major contributors to the growing burden of CKD over the past several decades (28–30). Although effective management of blood pressure and blood glucose levels plays a critical role in controlling the prevalence and progression of CKD, the control rates for both hypertension and diabetes in the province remain below the national average (31). This suboptimal management leading to inadequate control of blood pressure and blood glucose levels, and subsequent progression to CKD. Second, smoking, high-salt diet, and physical inactivity are well-established risk factors for chronic kidney disease (CKD) (32). These risk behaviors are highly prevalent in Yunnan Province (24), which likely contributes to the region’s high burden of CKD. However, most residents currently lack corresponding CKD intervention measures, highlighting an urgent need to strengthen CKD related health promotion efforts (15).

Previous studies have indicated that gender is a risk factor for CKD, with women generally exhibiting a higher prevalence than men (33). However, in our survey, no statistically significant difference was observed between males (12.7%; 95% CI: 10.1–15.8%) and females (13.5%; 95% CI: 10.2–17.6%). This finding may be attributed to the higher prevalence of hypertension and diabetes among males in Yunnan, potentially increasing their risk of developing CKD.

Advancing age is associated with an increased prevalence of CKD, consistent with findings from previous studies. This trend stems from two primary factors: Firstly, age-related physiological decline involves sclerosis of renal arteries and micro-vessels. Consequently, urinary protein excretion rises while the glomerular filtration rate (GFR) decreases. According to the current diagnostic criteria for CKD, this manifests as a higher prevalence rate among the older adult. Secondly, the older adult exhibit higher prevalence rates of comorbidities like hypertension and diabetes, which are significant pathological contributors to CKD development. However, the current CKD diagnostic criteria (1) are not age-adjusted. Consequently, establishing age-specific diagnostic criteria holds practical significance for distinguishing between physiological age-related renal decline and pathological renal impairment in the older adult population (34).

Additionally, this study found that educational level and marital status are associated with CKD prevalence. This association is largely attributable to the older age profile observed among individuals with lower education and those who are divorced, widowed, or separated, leading to a relatively higher CKD prevalence in these groups. Among patients with hypertension, diabetes, or hyperuricemia, CKD prevalence is significantly higher than in those without these conditions, aligning with findings from national surveys (35, 36). Hypertension, diabetes, and hyperuricemia are all established risk factors for CKD development and progression. Hypertension is present in up to 90% of CKD cases (37), while over 40% of diabetic patients develop chronic kidney disease (38). Hyperuricemia may trigger chronic kidney inflammation, resulting in renal function decline (39).

Previous studies indicate that 67% of chronic kidney disease (CKD) patients present with blood creatinine levels exceeding 177 μmol/L at diagnosis. Therefore, improving awareness and treatment is crucial to slowing down progression of CKD to end-stage renal disease. However, this study reveals alarmingly low rates in Yunnan Province: the CKD awareness is only 3.0%, and the treatment rate is 1.0%. This awareness is significantly lower than the national average (10.0%) (8) and findings from Shijingshan District, Beijing (7.3%) (7). Previous studies have found that among patients diagnosed with chronic kidney disease (CKD), approximately 75% of cases are attributed to infections, exposure to toxins (such as from animal bites, herbal remedies, or medications), or complications during pregnancy, which are often the circumstances leading to diagnosis (40). The low awareness and treatment rates are partly attributable to CKD’s insidious onset and lack of obvious early symptoms. However, the primary reason lies in insufficient societal understanding of CKD. While government, societal sectors, and individuals have increasingly focused on chronic diseases in recent years, the emphasis has predominantly been on four major conditions: cardiovascular and cerebrovascular diseases, chronic respiratory diseases, cancer, and diabetes (41). Consequently, investment and publicity efforts for CKD prevention and control remain relatively weaker compared to these priority diseases.

Media coverage of CKD is also limited especially in economically underdeveloped areas. In some remote mountainous regions of western China, residents often have relatively low health literacy, live in scattered communities, and have limited access to healthcare services. When confronted with illnesses that present subtle symptoms—such as chronic kidney disease, many lack sufficient health awareness or face financial constraints, which can prevent them to recognizing the severity of the condition.

Furthermore, in the management of patients with hypertension and diabetes though China national essential public health services, routine physical examinations do not include renal function or urine protein tests. This omission represents a key barrier to the timely identification of CKD complications among these patients. Compounding this issue is the insufficient attention some primary care providers pay to potential complications in their managed patients. In China, a vast number of patients are managed for hypertension and diabetes within the national essential public health services system. Leveraging these follow-up visits to integrate CKD interventions, such as screening, patient education, lifestyle guidance, and rational medication use, would help control relevant behavioral risk factors. In addition, cost-effective CKD risk prediction models should be incorporated into opportunistic screening strategies for various populations.

## 5 Limitations

This study has several limitations. First, CKD was defined solely by eGFR and urinary protein levels, without incorporating supplementary indicators such as serum cystatin C. This approach may have led to an underestimation of the true population prevalence. Second, the use of the CKD-EPI equation for eGFR estimation, while widely adopted, may overestimate CKD prevalence in Chinese populations due to ethnic-specific calibration limitations (42). Third, constrained by research design limitations, renal function parameters were assessed at a single time point, lacking confirmatory retesting after 3 months as recommended by clinical guidelines. Due to the incorporation of reversible conditions, such as acute kidney injury (AKI) and transient proteinuria, may compromise diagnostic accuracy and inflate the estimated prevalence rate. Fourth, due to the small sample size in certain subgroups-such as the Yi ethnic group, the precision of rate estimates is limited, as reflected in wider 95% confidence intervals. Fifth, as a cross-sectional study, it establishes associations between risk factors and CKD but cannot infer causality. The complex interplay of CKD determinants, including potential mediating effects and factor interactions requires prospective cohort studies for deeper exploration.

## 6 Conclusion

The findings of this study revealed that the estimated prevalence of chronic kidney disease (CKD) exceeds the national average (8.2%), among adults in Yunnan Province. Critically, CKD awareness and treatment remain alarmingly low. Therefore, implementing targeted early screening programs or community-based interventions be prioritized as urgent public health initiatives to improve disease recognition and treatment adherence for CKD prevention and management in China’s less developed regions.

## Data Availability

The raw data supporting the conclusions of this article will be made available by the authors, without undue reservation.
